# Monitoring lithium therapy in a CMHRS during the COVID-19 pandemic

**DOI:** 10.1192/bjo.2021.506

**Published:** 2021-06-18

**Authors:** Katherine Gardner, Charles Shuttleworth

**Affiliations:** Surrey and Borders Partnership NHS Foundation Trust

## Abstract

**Aims:**

The COVID-19 pandemic has presented a challenge in ensuring that routine monitoring can still be offered and occur in a safe and effective manner. Our aim was to continue the regular physical health monitoring of North East Hampshire CMHRS patients who are prescribed lithium during the COVID-19 pandemic, and to achieve above 90% compliance for the monitoring standards. Lithium monitoring clinics have been established in our CMHRS since 2017, Lithium is a high-risk medication as recognised by a National Patient Safety Alert in 2009. Previous annual POMH-UK audits have identified suboptimal monitoring of Lithium patients at local and national levels.

**Method:**

The ‘Plan-Do-Study-Act’ (PDSA) approach was utilised and a ‘QI Bundle’ formed. A database of patients who are prescribed lithium has been created and maintained. Synchronised standardised reminder letters are sent to the patients twice yearly. NICE recommends that patients on lithium have their lithium levels checked every 3 months for the first year and then at least every 6 months, plus TFTs, U&Es, calcium and weight every 6 months (or more frequently if impaired). The Specialist Pharmacist Service advise that during the COVID-19 pandemic, if patients are not in the at-risk category then monitoring intervals can be extended by up to 3 months, but that patients in the at-risk category should have their normal monitoring intervals continued.

Lithium clinics have been held every April and October since 2017 by the Junior Doctors allocated to NE Hampshire CMHRS. This year they were conducted via telephone appointment or face to face where safe to do so with clinician in full PPE. An audit was subsequently carried out in December 2020 to assess our patient's compliance with the aforementioned NICE recommendations for lithium monitoring, the results of which were compared to previous annual audits.

**Result:**

Seventeen patients were currently prescribed lithium within the NE Hampshire CMHRS. Over 90% compliance with monitoring was achieved in December 2020 apart from the checking of calcium levels which was slightly below target at 82%, and weight which was at 88%. There was no significant reduction in monitoring standards obtained compared to data from the previous three years.

**Conclusion:**

Routine monitoring for our patients who are prescribed Lithium was effectively and safely continued during the pandemic. Above 90% compliance with lithium monitoring standards was nearly obtained across all areas. We will continue to offer lithium monitoring twice yearly.

